# Supplementary figures and images for: Dynamic bistable switches enhance robustness and accuracy of cell cycle transitions
Source: PLoS Comput Biol. 2021 Jan 7;17(1):e1008231. doi: 10.1371/journal.pcbi.1008231 (PMC7817062; doi:10.1371/journal.pcbi.1008231)

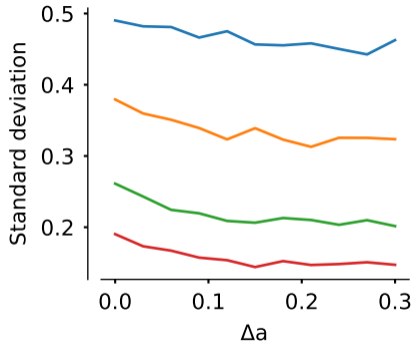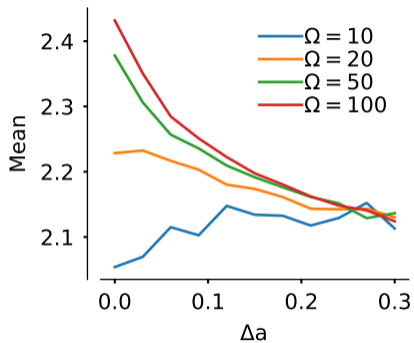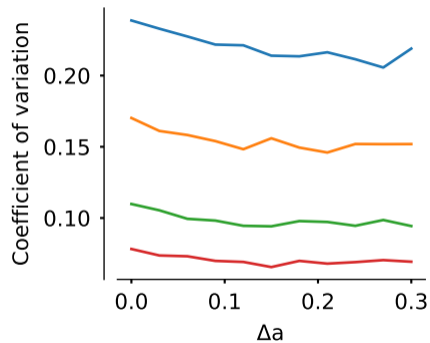

Supplement: S1 Fig — (PDF) [file pcbi.1008231.s001.pdf]

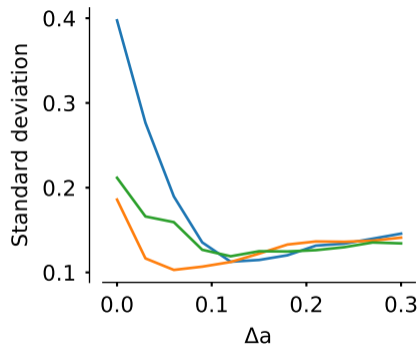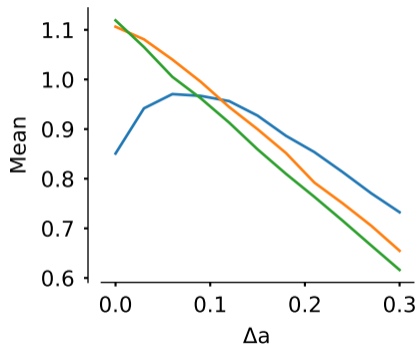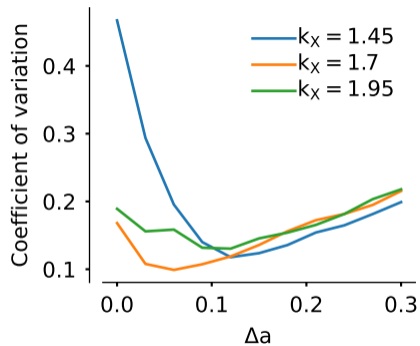

Supplement: S2 Fig — (PDF) [file pcbi.1008231.s002.pdf]

$k_X = 1.45$ 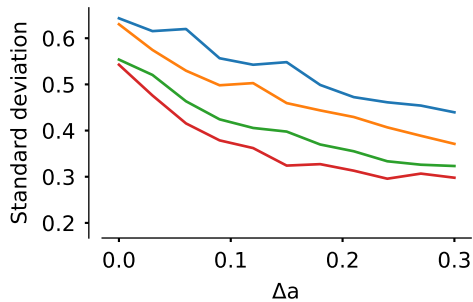 $k_X = 1.7$ 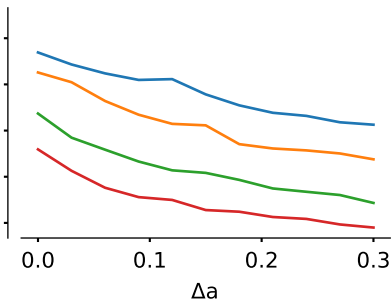 $k_X = 1.95$ 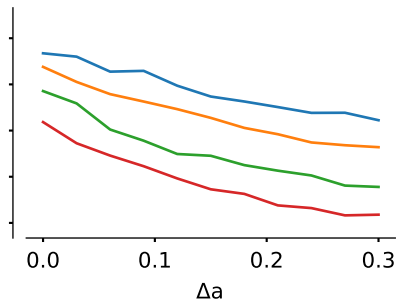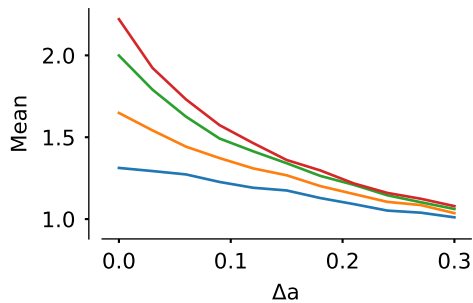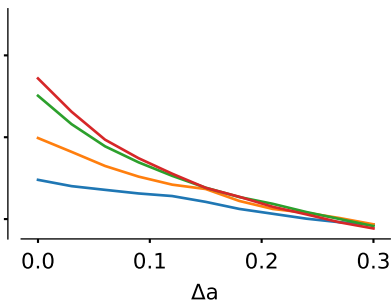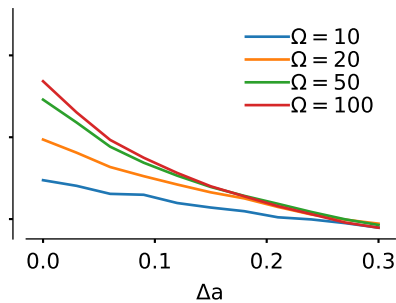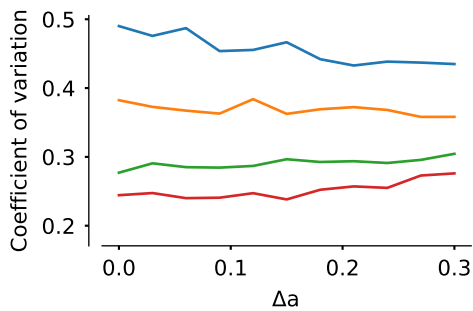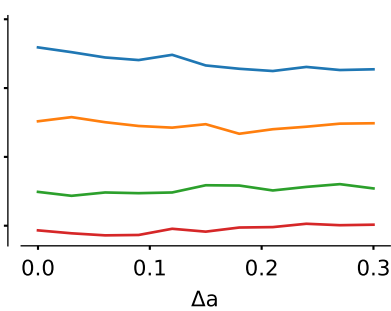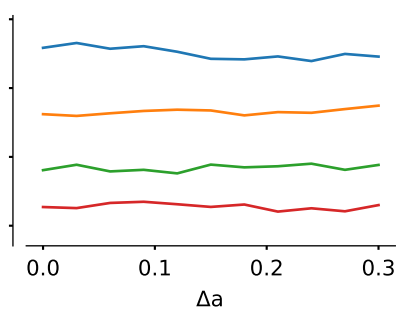

Supplement: S3 Fig — (PDF) [file pcbi.1008231.s003.pdf]

$\tau = 0.0, \kappa = 1.0$ 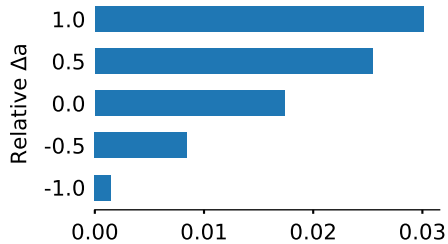 $\tau = 0.0, \kappa = 5.0$ 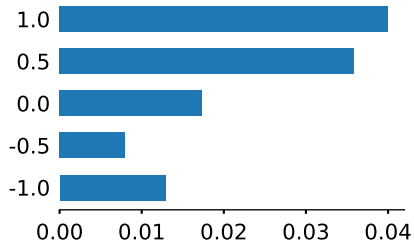 $\tau = 0.0, \kappa = 10.0$ 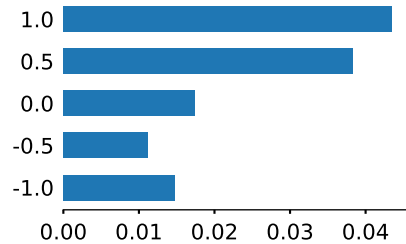 $\tau = 0.1, \kappa = 1.0$ 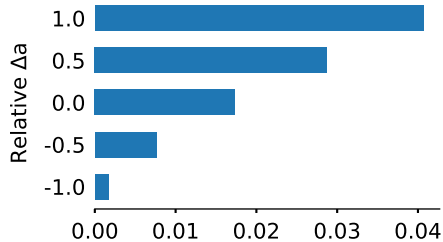 $\tau = 0.1, \kappa = 5.0$ 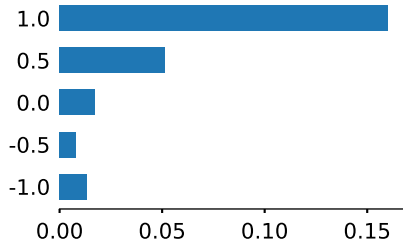 $\tau = 0.1, \kappa = 10.0$ 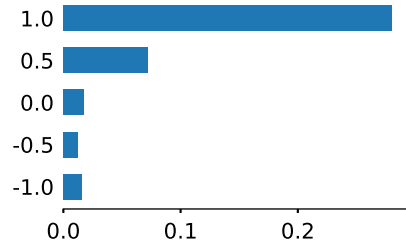 $\tau = 0.2, \kappa = 1.0$ 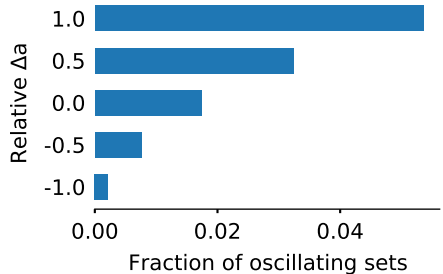 $\tau = 0.2, \kappa = 5.0$ 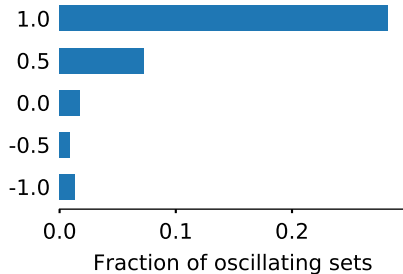 $\tau = 0.2, \kappa = 10.0$ 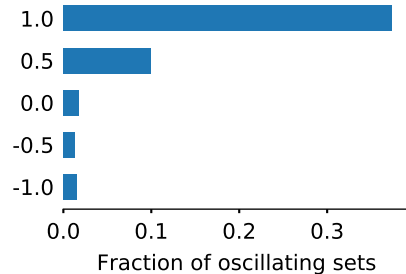

Supplement: S4 Fig — (PDF) [file pcbi.1008231.s004.pdf]
