# Supplementary material for: Dynamic bistable switches enhance robustness and accuracy of cell cycle transitions
Source: PLoS Comput Biol. 2021 Jan 7;17(1):e1008231. doi: 10.1371/journal.pcbi.1008231 (PMC7817062; doi:10.1371/journal.pcbi.1008231)
Supplement: S1 Table — All parameters except for the import and export rates and ks were taken from [60]. (PDF) [file pcbi.1008231.s005.pdf]

Symbol	Meaning	Value
k_s	Cyclin production rate	1.5 nM/min
a_{Cdc25}	Basal Cdc25 activity	0.16
b_{Cdc25}	Maximal Cdc25 activity	0.8
K_{Cdc25}	Threshold for Cdc25 activation	35 nM
m_{Cdc25}	Hill exponent for Cdc25 activation	11
a_{Wee1}	Basal Wee1 activity	0.08
b_{Wee1}	Maximal Wee1 activity	0.4
K_{Wee1}	Threshold for Wee1 activation	30 nM
m_{Wee1}	Hill exponent for Wee1 activation	3.5
$k_{n,\mathrm{Cyc}}$	Cyclin B-Cdk1 import rate into nucleus (unmodified)	0.05 1/min
$k_{c,\mathrm{Cyc}}$	Cyclin B-Cdk1 export rate from nucleus	0.05 1/min
$k_{n,\mathrm{Cdc25}}$	Cdc25 import rate into nucleus (unmodified)	0.05 1/min
$k_{c,\mathrm{Cdc25}}$	Cdc25 export rate from nucleus	0.05 1/min
