# Supplementary material for: Dynamic bistable switches enhance robustness and accuracy of cell cycle transitions
Source: PLoS Comput Biol. 2021 Jan 7;17(1):e1008231. doi: 10.1371/journal.pcbi.1008231 (PMC7817062; doi:10.1371/journal.pcbi.1008231)
Supplement: S2 Table — For ε, we sampled the logarithm: we sampled a number γ between ln(0,01) and ln(1) uniformly and then took ε = eγ. (PDF) [file pcbi.1008231.s006.pdf]

Parameter	Low	High	Logarithmic?
a	0.01	2	No
a'	0.01	2	No
b	0.01	10	No
b'	0.01	10	No
K	0.01	5	No
K'	0.01	5	No
n	1	15	No
m	1	15	No
k_X	0.1	5	No
ϵ	0.01	1	Yes
