# Supplementary material for: Dynamic bistable switches enhance robustness and accuracy of cell cycle transitions
Source: PLoS Comput Biol. 2021 Jan 7;17(1):e1008231. doi: 10.1371/journal.pcbi.1008231 (PMC7817062; doi:10.1371/journal.pcbi.1008231)
Supplement: Attachment — Submitted filename: Review_Rombouts&Gelens.pdf [file pcbi.1008231.s014.pdf]

The manuscript "Dynamic bistable switches enhance robustness and accuracy of cell cycle transitions" investigates the role of a time-varying dynamic bistable switch in cell-cycle progression using a conceptual mathematical model. The authors demonstrated that the dynamic bistable switch, which could arise from the compartmentalization in intracellular space, could lead to accurate and robust mitotic entry transition timing. Furthermore, the authors also illustrated that the dynamic switch could lead to traveling fronts and stable oscillations of protein activity and abundance. Considering the impact of the result, I believe that the manuscript fits well with PLoS Computational Biology. However, several points listed below should be clarified before publications.

Major comments

1. For the stochastic simulation in the Fig 4 and 5, the authors used Langevin type equations with noise only in the fast variable as the noise level of fast variable is expected to be stronger than the noise level of slow variable However, even this is true, the noise of slow variable can be amplified via its effect on the fast variable (i.e., slow and fast variables are interacting, which is the major focus of this manuscript). As the major conclusion of the manuscript is "dynamical switch, which is based on the interaction between slow and fast variables, induces more stable transition timing (Fig. 4) and oscillation (Fig. 5)", the stochastic simulations for these interactions should be performed. We understand that the original Gillespie algorithm for mass action kinetics cannot be performed. However, the Gillespie algorithm with non-elementary propensity functions of the model can be alternative (e.g. Kim, Josic, Bennett, BMC Syst Biol, 2015). Specifically, Gillespie algorithm can be performed for the transition time with the three non-elementary propensity functions, f(X)(X_T-X)/epsilon, g(X)X/epsilon, and k_X.

2. The figure 5F and G show that the oscillation of the protein X abundance is maintained even if the value of Δa becomes negative. Then, when its value is smaller, the oscillatory dynamics disappears and occurs again. Please clearly describe why the oscillatory dynamics is maintained even if the value of Δa is negative (i.e. the bistable threshold moves to the right when the total concentration of X approaches it). Furthermore, describe the reappearance of the dynamics.

Minor comments

1. Referring the set of equations for the biological example in Section 4.6 of Materials and Methods (i.e. Eqs 14-23), the active Cyclin B-Cdk1 complex is produced at the rate of k_s in the cytoplasm while the inactive complex is not produced (i.e., the production rate of the active Cyclin B-Cdk1 complex in the cytoplasm is same to that of the total Cyclin B-Cdk1 complex in the cytoplasm (see Eqs. 15 and 19), indicating that the production rate of the inactive Cyclin B-Cdk1 complex in the cytoplasm is zero). Please describe biological rationale giving this restriction. If this is just a typo, please revise this.

2. To test the robustness and the accuracy of the oscillatory system, the authors solely focused on the coefficient of variation of period (fig 5E). It would be nice to test whether the amplitude becomes more stable when the value of Δa increases (optional).

3. Please add references for the following sentence in Page 5 Line 177, "This example can be

extended by including nuclear envelope breakdown (NEBD). Cdk1 activation triggers this event (Reference), which effectively mixes the two compartments."

4. The authors mentioned that spatial compartmentalization has recently been a topic of interest for mathematical modelers in Discussion (Page 12 Line 409). To support this, please add references from various areas. Exemplary references for this are circadian rhythms of p53 (Gotoh et al., PNAS, 2016 and Xianlin et al., Front in Physiol, 2020), circadian rhythms of CRY (Yoo et al, Cell 2013).

4. The authors mentioned that rigorous mathematical analysis of multiple time-scale dynamical systems, such as the model used in this study, can provide a valuable insight into our current understanding of biological systems in Discussion (Page 13 Line 462). However, we believe the references for multi-scale stochastic systems would be more relevant as the major topic of the manuscript is the role of dynamic switch against stochastic noise. Here are a couple references for the analysis and simulations of multi-scale stochastic simulations (Rao and Arkin, JCP (2003), Schnoerr D et al, JPA (2017), Kim and Sontag, PLOS Com (2017)).

5. Typo in Page 6 fig3 legend: "The vertical orange curve, when followed from bottom to top, corresponds to the orange response curve Panel B." → "The vertical orange curve, followed from bottom to top, corresponds to the orange response curve in Panel B."

6. Typo in Page 15 Line 548: "We keep track of three different variables: total Cyclin B-Cdk1 complexes ([Cyc], active …" → "We keep track of three different variables: total Cyclin B-Cdk1 complexes ([Cyc]), active …"
