# Supplementary material for: Dynamic bistable switches enhance robustness and accuracy of cell cycle transitions
Source: PLoS Comput Biol. 2021 Jan 7;17(1):e1008231. doi: 10.1371/journal.pcbi.1008231 (PMC7817062; doi:10.1371/journal.pcbi.1008231)
Supplement: Attachment — Submitted filename: responsetoreviewers.pdf [file pcbi.1008231.s015.pdf]

Response to reviewers' comments
Dynamic bistable switches enhance robustness and accuracy of cell cycle transitions

Jan Rombouts and Lendert Gelens

October 26, 2020

Reviewer #1

Reviewer: This is nice work that is entirely suitable for publication in PLoS Comp Biol.

The authors have already made most of the changes that I recommended in an earlier review of the paper for a different journal.

> **Authors:** We are glad the reviewer likes our paper and that he/she appreciates the changes we have made in response to the earlier comments.

Reviewer: A necessary revision: the authors should create a table that lists all the parameter values used in the calculations in the figures. For example, I wanted to check Fig. 3, and it took me a little bit of trial-and-error to figure out the parameter values used for the model in this figure.

> **Authors:** The parameter values for Fig. 3 are the same are those used in Fig. 4 and were mentioned in the captions. We agree that the values are too hard to find, so we include a table in the methods section with the standard parameter set used.

Reviewer: An optional revision: In the Discussion, the authors say "It would be valuable to study a model of the form... Eq. (7)". They don't have to put this off to a future publication, because they are already set up to do so using the two-param bifurcation diagram in Fig 3D. In the attached file, I present an implementation of Eq. (7) that produces limit cycle oscillations. When XT(t) and a(t) are projected onto Fig. 3D, the limit cycle sweeps diagonally across the bistable domain (the blue region), alternately flipping X(t) into the high and low states of the bistable switch. It would be a nice example of how to analyze a system with time-scale separations.

Authors: We thank the reviewer for this suggestion and the attachment. We agree this is a nice way to show what may be the effect of different timescale separations between the three variables. We now introduce a model similar to the one proposed by the reviewer in the Discussion, and added a panel to Fig. 5 (Panel L) showing the effect of different timescales in a projection of the limit cycle on the (a, X_T) plane as suggested. We also include an animation (S6 Video) to show the behavior for different timescales.

Reviewer #2

Reviewer: The idea presented in this manuscript is very interesting, but I think it can be improved:

Authors: We are happy the reviewer thinks our work is very interesting. Below we reply to his/her suggestions for improvement.

Reviewer: - The introduction is broad and it doesn't focus on the main ideas stated in the abstract. It would be nicer to have more details about dynamical stability than explain bistable switches in the cell cycle... which they have been broadly covered.

Authors: We are not entirely sure what the reviewer means, but we interpret it as saying that it would be nice to have more explanation of the dynamical role of bistable switches in biological transitions and oscillations. We have added more along these lines to the Introduction and we have significantly expanded and restructured our Discussion and Conclusion section.

Reviewer: - At the end of section 2.1, there is explained that the NEBD is another example, but it is not supported by any analysis or bibliography covering the dynamical changes on bistable switches when including NEBD.

Authors: We think NEBD is indeed an example of how bistable response curves change over time, but this has, as far as we know, not been studied using this interpretation, hence the lack of bibliography in this part. We mention the NEBD, together with translocation of other proteins such as Greatwall, as another example of a situation with changing switches which could potentially be analyzed similarly as our analysis of mitotic entry here. We think a full analysis of these situations with a more detailed mathematical model deserves a more in-depth study out of scope for the current paper. We have changed the text to make our intentions more clear.

Reviewer: - Fig 2C. It is difficult to follow together with the description in the main test. It would be nice to have some kind of guidance when walking through the different plots within the test. Besides, the colours are misleading, as one can think that blue

curve is the complex cycB-CDK1 and Cdc25 is red (based on the diagram on Fig 2A). Choose another colour scheme would be helpful.

Authors: We agree that this picture is hard to follow. In addition to the animation which we had already included in the first version of the manuscript, we have now updated this picture. We have added a panel with a time axis showing the evolution of Cdk1 activity in nucleus and cytoplasm, together with indications of the time points at which the snapshots of the bistable response curve were taken. We have also changed the color scheme as suggested.

Reviewer: - Section 2.2. seems completely irrelevant for the purpose of the manuscript. The system explained here can de added into the next 2 sections, as it seems to be the original work described in the manuscript.

Authors: We have removed this subtitle and merged the section with the next one.

Reviewer: - Apart from section 2.1, there is no extra connection with cell cycle, either in the switches or the oscillatory behaviour. The toy examples used to explain the idea are quite useful, but I am missing a relation between these examples and the real players of the cell cycle (out of the brief explanation on the discussion).

Authors: We have, at various points in the manuscript, made a more direct link between our toy model variables X_T and X and the proteins Cyclin B and Cdk1, which are the main players in the switch at the G2/M transition. In the discussion, we have added a paragraph describing how some of the results of our toy model may apply to other switches in the cell cycle. We do not make a direct mapping from the toy model variables to these other switches here, because the molecular mechanisms do not directly correspond.

Reviewer: - The title of the work oversells the content of the manuscript. If kept, I will consider to make a strong point based on the previous comment.

Authors: We have decided to keep the title as it is and hope that our adaptations in response to the previous point are sufficient to justify this.

Reviewer #3

Reviewer: The manuscript "Dynamic bistable switches enhance robustness and accuracy of cell cycle transitions" investigates the role of a time-varying dynamic bistable switch in cell-cycle progression using a conceptual mathematical model. The authors demonstrated that the dynamic bistable switch, which could arise from the compartmentalization in intracellular space, could lead to accurate and robust mitotic entry

transition timing. Furthermore, the authors also illustrated that the dynamic switch could lead to traveling fronts and stable oscillations of protein activity and abundance. Considering the impact of the result, I believe that the manuscript fits well with PLoS Computational Biology. However, several points listed below should be clarified before publications.

Authors: We are glad the reviewer thinks our work is suitable for the journal. We address the points he/she raises below.

Reviewer: Major comments 1. For the stochastic simulation in the Fig 4 and 5, the authors used Langevin type equations with noise only in the fast variable as the noise level of fast variable is expected to be stronger than the noise level of slow variable However, even this is true, the noise of slow variable can be amplified via its effect on the fast variable (i.e., slow and fast variables are interacting, which is the major focus of this manuscript). As the major conclusion of the manuscript is "dynamical switch, which is based on the interaction between slow and fast variables, induces more stable transition timing (Fig. 4) and oscillation (Fig. 5)", the stochastic simulations for these interactions should be performed. We understand that the original Gillespie algorithm for mass action kinetics cannot be performed. However, the Gillespie algorithm with non-elementary propensity functions of the model can be alternative (e.g. Kim, Josic, Bennett, BMC Syst Biol, 2015). Specifically, Gillespie algorithm can be performed for the transition time with the three non-elementary propensity functions, f(X)(X_T-X)/epsilon, g(X)X/epsilon, and k_X.

Authors: We have performed stochastic simulations both of the transition timing and of the oscillatory system which includes degradation. We added panels to Figures 4 and 5 describing the results. The results in general point to the same conclusion as our simulations of the Langevin equations, however, the effect is much smaller. For the transition timing (Figure 4), we had to simulate the system 2000 times instead of 200 times, which was our standard sample size for the Langevin equation, to see a discernible decrease of the coefficient of variation. The decrease is visible for low molecule numbers, which corresponds to larger noise strengths. A similar result holds for the period in the oscillating system.

The results for the stochastic simulations are thus less clear, but we believe that our general conclusion is still warranted because of the following points: a) If we look at standard deviation instead of CV, the effect is clearer and much smoother. In the main figure we have kept the CV to be in line with our previous plots and because, since we compare the variation of distributions with different means, we consider it more correct to compare the CV. However, this shows that it depends on which measure of variation is taken. We have now added extra figures in the Supporting Information (Figures S1 and S3) which also show how the standard deviation and the mean of transition timing and period change with switch variability. b) The dependence of the switch shape depends directly on the variable X_T, which is noisy

in the fully stochastic version. Therefore, the switch variation will also be noisy which in part reduces the stabilizing effect the switch change has on the period. We think this is a limitation of our artificial implementation of the switch shape change. Other implementations may lead to a more robust result.

We have added these caveats to the text of the article, with a reference to the supplemental figures.

Reviewer: 2. The figure 5F and G show that the oscillation of the protein X abundance is maintained even if the value of Δa becomes negative. Then, when its value is smaller, the oscillatory dynamics disappears and occurs again. Please clearly describe why the oscillatory dynamics is maintained even if the value of Δa is negative (i.e. the bistable threshold moves to the right when the total concentration of X approaches it). Furthermore, describe the reappearance of the dynamics.

Authors: Oscillations for small negative values of Δa can be explained by the fact that if the threshold moves to the right as the system approaches it, the system can still manage to cross the threshold if the production rate k_X is sufficiently high. These oscillations are thus "of the same type" as the oscillations in the region $\Delta a > 0$.

The reappearance of oscillations when Δa takes on more negative values is less easy to explain, and is best seen as an artefact of our implementation of the varying switch. Since we model the switch variation using the formula

$$a = \bar{a} + \Delta a \tanh(\kappa(X_T - X_c)),$$

if Δa is more negative, this means that a is higher initially, for low values of X_T. This means that the activation threshold lies more to the left. In the more extreme cases, the threshold lies so far to the left that the system can cross it before the switch variation given by the formula above brings this threshold to the right. Note that the oscillations in this case are of smaller amplitude, and the switch stays on the left.

We have added a supplemental animation (S5 Video) to explain this. We have also added an additional remark in the main text about this reappearance of oscillations.

Reviewer: Minor comments 1. Referring the set of equations for the biological example in Section 4.6 of Materials and Methods (i.e. Eqs 14-23), the active Cyclin B-Cdk1 complex is produced at the rate of ks in the cytoplasm while the inactive complex is not produced (i.e., the production rate of the active Cyclin B-Cdk1 complex in the cytoplasm is same to that of the total Cyclin B-Cdk1 complex in the cytoplasm (see Eqs. 15 and 19), indicating that the production rate of the inactive Cyclin B-Cdk1 complex in the cytoplasm is zero). Please describe biological rationale giving this restriction. If this is just a typo, please revise this.

Authors: Indeed, the production of active complex is the same as the production

as total complex. We base our model on the model introduced by Yang and Ferrell (2013), which assumes that a) free Cdk1 concentrations are high compared to typical Cyclin B concentrations, b) Cyclin B has a high affinity for Cdk1, such that all produced cyclin immediately binds to a Cdk1 partner and c) that the newly formed Cdk1-Cyclin B complexes are immediately active. These assumptions have as net effect that all produced Cyclin B directly produces active Cdk1-Cyclin B, which explains the appearance of the k_s term in both equations.

We extended the explanation in the text to clarify.

Reviewer: 2. To test the robustness and the accuracy of the oscillatory system, the authors solely focused on the coefficient of variation of period (fig 5E). It would be nice to test whether the amplitude becomes more stable when the value of Δa increases (optional).

Authors: We have also measured the variation of the amplitude. A little variation in the switch decreases amplitude variation, but this saturates and for larger switch variations, the variation even slightly increases. However, it is still clear that a varying switch produces less variation than a static switch.

We decided not to include this result in a main figure, but we have added a supplemental figure (Figure S2) with these results and a sentence in the main text explaining the result and refering to the figure.

Reviewer: 3. Please add references for the following sentence in Page 5 Line 177, "This example can be extended by including nuclear envelope breakdown (NEBD). Cdk1 activation triggers this event (Reference), which effectively mixes the two compartments."

Authors: We have added a reference to a recent review paper (Ungricht and Kutay, Nat. Rev. Mol. Cell Biol. 2017) which describes the mechanisms underlying nuclear envelope breakdown.

Reviewer: 4. The authors mentioned that spatial compartmentalization has recently been a topic of interest for mathematical modelers in Discussion (Page 12 Line 409). To support this, please add references from various areas. Exemplary references for this are circadian rhythms of p53 (Gotoh et al., PNAS, 2016 and Xianlin et al., Front in Physiol, 2020), circadian rhythms of CRY (Yoo et al, Cell 2013).

Authors: We agree that the influence of nucleocytoplasmic transport in other systems should be included in our discussion at this point, and thank the reviewer for the suggestions. We have extended the discussion accordingly.

Reviewer: 4. The authors mentioned that rigorous mathematical analysis of multiple time-scale dynamical systems, such as the model used in this study, can provide a valu-

able insight into our current understanding of biological systems in Discussion (Page 13 Line 462). However, we believe the references for multi-scale stochastic systems would be more relevant as the major topic of the manuscript is the role of dynamic switch against stochastic noise. Here are a couple references for the analysis and simulations of multi-scale stochastic simulations (Rao and Arkin, JCP (2003), Schnoerr D et al, JPA (2017), Kim and Sontag, PLOS Com (2017)).

Authors: We have added this aspect to the discussion.

Reviewer: 5. Typo in Page 6 fig3 legend: "The vertical orange curve, when followed from bottom to top, corresponds to the orange response curve Panel B." → "The vertical orange curve, followed from bottom to top, corresponds to the orange response curve in Panel B."

Authors: Fixed, thank you.

Reviewer: 6. Typo in Page 15 Line 548: "We keep track of three different variables: total Cyclin B-Cdk1 complexes ([Cyc], active ..." → "We keep track of three different variables: total Cyclin B- Cdk1 complexes ([Cyc]), active ..."

Authors: Fixed, thank you.
