# Supplementary material for: Dynamic bistable switches enhance robustness and accuracy of cell cycle transitions
Source: PLoS Comput Biol. 2021 Jan 7;17(1):e1008231. doi: 10.1371/journal.pcbi.1008231 (PMC7817062; doi:10.1371/journal.pcbi.1008231)
Supplement: Attachment — Submitted filename: responsetoreviewers.pdf [file pcbi.1008231.s016.pdf]

Response to reviewers' comments
Dynamic bistable switches enhance robustness and accuracy of cell cycle transitions

Jan Rombouts and Lendert Gelens

November 16, 2020

Reviewer #3

Reviewer: The authors have addressed most of concerns in the revision. However, according to the new result, the description for the main results needs to be more clear. The authors newly performed the stochastic simulations for the case when the slow variable also fluctuates (Fig 4I, J, Fig 5G, and Fig S1 and S3). According to these new results, the advantage of dynamic switch overs the static switch is not clear. Even dynamic switch leads to more unstable oscillations than the static switch when Omega=50 and 100 (e.g. red and green line; Fig S3 left lowest panel). Thus, the current results indicate that the dynamic switch leads to robustness (stability) over the static switch "under the condition that the noise of the slow variable, which leads to the dynamic switch (i.e. XT), is negligible". However, such limitation seems not clearly descried in the current text including intro and conclusion. Providing the clear condition when the dynamic switch is robust will improve the impact of the manuscript and be more helpful for readers when they want to use the results.

Authors: The reviewer is right in pointing out that, in the stochastic simulation algorithm, the effect of the dynamic switch is not that large and even absent in some conditions. We had already mentioned some possible explanations in the text and we believe that to fully understand the effect, a more detailed model might be useful. In such a model the dynamic switch would arise from biochemical interactions and not artificially using a direct function of X_T, as here. We fully agree with the reviewer that this should be pointed out in the text. We have adapted the text in different locations. In the introduction and conclusion, we now mention that the effect of the dynamic switch in noisy systems is conditional on the size of the noise. In the section on the transitions, we elaborate a bit and also mention that in a more detailed model, this requirement may be relaxed.
