# Supplementary material for: Dynamic bistable switches enhance robustness and accuracy of cell cycle transitions
Source: PLoS Comput Biol. 2021 Jan 7;17(1):e1008231. doi: 10.1371/journal.pcbi.1008231 (PMC7817062; doi:10.1371/journal.pcbi.1008231)
Supplement: Attachment — Submitted filename: Gelens.docx [file pcbi.1008231.s013.docx]

#Gelens

F(x,a,b,K,n) = a + b*x^n/(K^n+x^n)

G(x,a,b,K,n) = a + b*K^n/(K^n+x^n)

dX/dt = (F(X,a,b,K,n)*(XT-X) - G(X,ap,bp,Kp,m)*X)/eps

\# dXT/dt= k0 - XT

dXT/dt= k0 - X*XT

da/dt = kap*( F(XT,a0,2*a1,Xc,p) - a )

\# par a=.15

par b=1, K=1, n=4, eps=.01

par ap=0, bp=1.3, Kp=1, m=4

par k0=2

par a0=.1, a1=.2, Xc=2.5, p=4, kap=10

done
